# Magnetic resonance imaging of intracranial hemangiopericytoma and correlation with pathological findings

**DOI:** 10.3892/ol.2014.2503

**Published:** 2014-09-04

**Authors:** CONG MA, FENG XU, YU-DONG XIAO, RAMCHANDRA PAUDEL, YI SUN, EN-HUA XIAO

**Affiliations:** 1 Department of Radiology, The Second Xiangya Hospital of Central South University, Changsha, Hunan 410011, P.R. China; 2 Yinzhou People’s Hospital, Ningbo, Zhejiang 315000, P.R. China; 3 Department of Pathology, The Second XiangYa Hospital of Central South University, Changsha, Hunan 410011, P.R. China

**Keywords:** HPC, intracranial, MRI, immunohistochemical

## Abstract

The present study aimed to evaluate the radiological and pathological features of intracranial hemangiopericytoma, and improve the understanding of this tumor. A retrospective analysis of radiological and pathological features of five cases of intracranial hemangiopericytoma was conducted between 2006 and 2012 in the Second Xiangya Hospital of Central South University. A total of five cases (three males and two females; aged 37–60 years) were enrolled. Magnetic resonance imaging revealed that the lesions were lobulated with iso-intensity T1-weighted image signals and slightly long T2-weighted image signals. Cystic degeneration, necrosis and flow void were observed. The case with the lesion located under the tentorium cerebelli exhibited compression of the fourth ventricle with lateral ventricle dilatation hydrocephalus. In all cases, the solid section of the lesion was markedly enhanced following injection of the contrast agent, and intratumoral vessels were observed. No case exhibited the dural tail sign. Immunohistochemical examination revealed positive expression of cluster of differentiation 34(CD34), vimentin and CD99, and negative expression of epithelial membrane antigen, S100 and glial fibrillary acidic protein. Proliferating cell nuclear antigen Ki-67 immunohistochemical staining revealed that <5% of cells expressed Ki-67 in two cases and 5–10% of cells expressed Ki-67 in three cases. In conclusion, intracranial hemangiopericytoma exhibits certain distinctive characteristics in radiological examination, allowing for improved diagnosis. However, pathological examination is required for confirmation.

## Introduction

Intracranial hemangiopericytoma (HPC) is a rare tumor well-known for clinically aggressive behavior in growth and infiltration. HPC accounts for 0.4% of all primary central nervous system tumors (1). HPC is considered to arise from the pericytes of the capillaries and has certain similar features to other types of tumor (2). The 2007 World Health Organization (WHO) classification has divided intracranial HPC into two separate categories: WHO Grade II HPC and WHO Grade III anaplastic HPC (3). Surgical resection is the standard treatment for HPC, however, the procedure presents a challenge as it may lead to extensive blood loss (4). Previosu studies have indicated that tumor recurrence is common in HPC patients and thus, radiotherapy is used to treat recurrent HPC (5). The radiological appearance of HPC resembles that of meningioma, but the pathological features resemble those of solitary fibrous tumors (6). The present study describes five cases of intracranial HPC diagnosed by pathology with the aim of comparing the radiological and pathological features.

## Materials and methods

### Patient information

A total of five pathologically proven cases of intracranial HPC were collected between May 2006 and March 2012 in the Second Xiangya Hospital of Central South University (Changsha, China). Of the five cases, four were classified as WHO Grade II and one was classified as WHO Grade III. All cases had undergone magnetic resonance imaging (MRI) examination. The images were evaluated by two radiologists and the final diagnosis was confirmed following evaluation of the specimens by two pathologists. The procedures followed in the present study were in accordance with the ethics committee of the Second Xiangya Hospital of Central South University and written informed consent was obtained from all patients. All the cases were followed up for between one and seven years, and each case enrolled in this study was kept anonymous following the retrieval of the follow-up information.

### MRI

GE Signa 1.5 T superconducting MRI (GE Healthcare, Little Chalfont, USA) was performed using a standard head coil, with a thickness of 5 mm and a layer distance of 1.5 mm, using spin-echo T1-weighted image [T1WI; repetition time (TR), 400–500 msec; echo time (TE), 15–30 msec] and fast spin-echo T2WI (TR, 3,000–4,500 msec; TE, 70–120 msec). Gadolinium diethylenetriamine penta-acetic acid (Gd-DTPA) contrast agent was adopted at dose, 0.1 mmol/kg; injection flow rate, 3 ml/sec; and scan parameter, fat suppression Flair T1WI (TR, 2,000–2,500 msec; TE, 7–13 msec).

### Pathological and immunohistochemical analysis

All cases underwent total resection of the tumor. The specimen was fixed in 4% neutral formalin, dehydrated and embedded in paraffin. Subsequently the sample was cut into 2.5 μm slices and underwent routine hematoxylin-eosin staining and immunohistochemical analysis of cluster of differentiation 34 (CD34), CD99, vimentin (Vim), S-100, epithelial membrane antigen (EMA), glial fibrillary acidic protein (GFAP), Ki-67 and synapsin (Syn) (Maxin-Bio, Co., Fuzhou, China) expression.

## Results

### Clinical findings

A total of five cases (three males and two females; age range, 37–60 years) were enrolled. Headache (n=5) and dizziness (n=4) were the most common presenting symptoms, followed by vomiting (n=2), weakness (n=1) and blurred vision (n=1). The routine laboratory findings were non-specific. During the follow-up period, one case recurred within four years of tumor resection; however, no cases developed metastases during the follow-up period. The clinical findings of each case are listed in Table I.

### MRI findings

**Figure 1:**
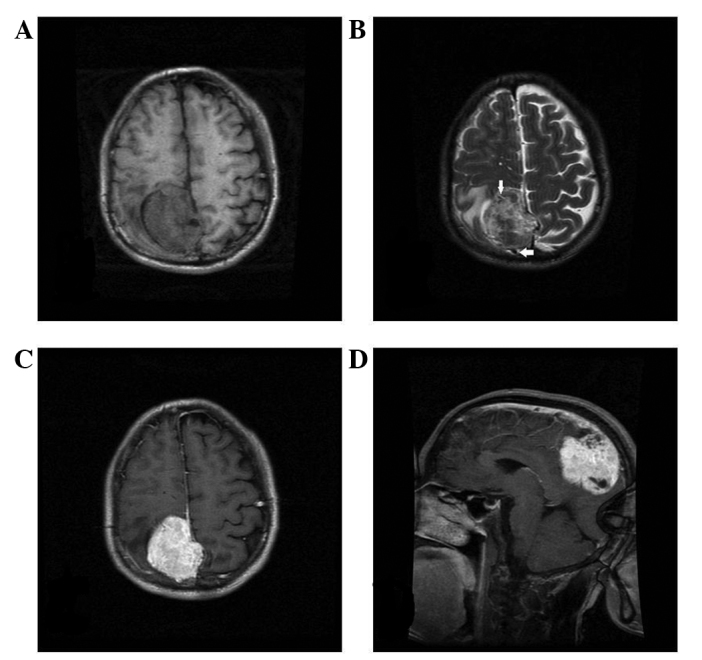
Patient 1. (A) Unenhanced magnetic resonance imaging scan reveals a heterogeneous iso-signal intensity mass in the right occipital region with a well-defined border. (B) The lesion has a marginally long T2 signal with certain intratumoral vessels (arrow), and peritumoral brain edema may be observed. (C) Enhanced scan demonstrates that the mass was markedly heterogeneously enhanced. (D) Cystic degeneration, necrosis and flow void are visible.

**Figure 2:**
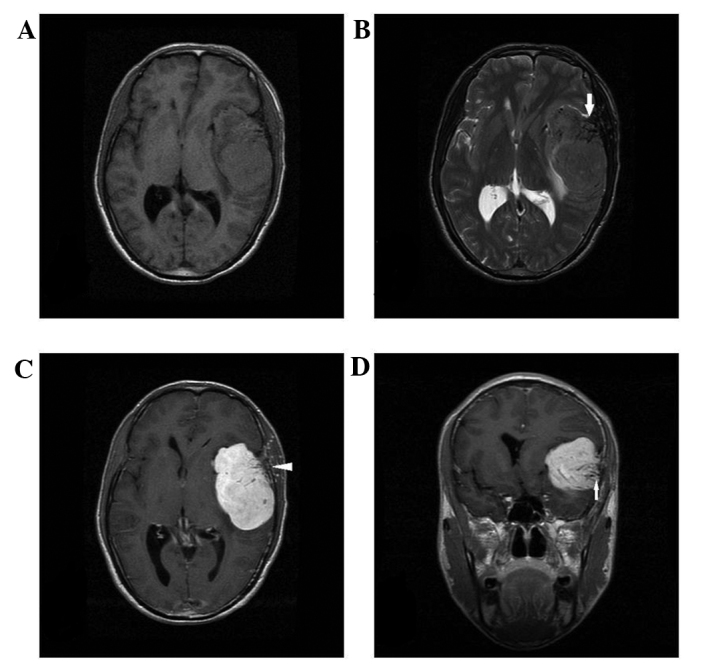
Patient 2. (A) Unenhanced magnetic resonance imaging scan reveals a mass in the left temporal region with heterogeneous iso-signal intensity in the T1-weighted image, and (B) heterogeneous marginally high signal intensity with numerous irregular intratumoral vessels (arrow) in the T2-weighted image. (C) Enhanced scan reveals that the mass is markedly enhanced. The adjacent bone exerts an obstruction effect due to the invasive growth pattern of the tumor (triangle). (D) Coronal reconstruction indicates that the flowing void effect may be visible (arrow).

**Figure 3:**
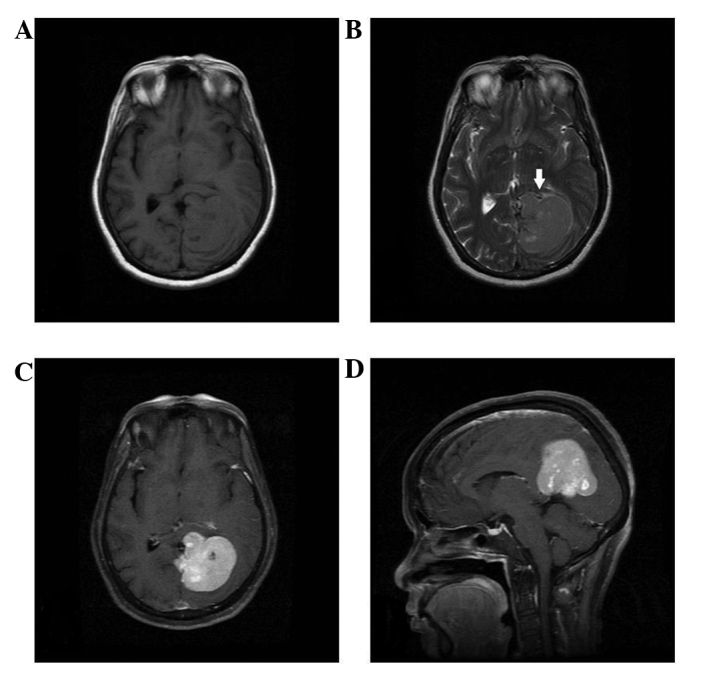
Patient 3. (A) Unenhanced magnetic resonance imaging scan reveals that the boundary of the tumor is well defined and lobular in shape. (B) Flow void can be observed in the T2-weighted image. (C and D) The lesion is markedly enhanced and the dural tail sign is not detectable.

**Figure 4:**
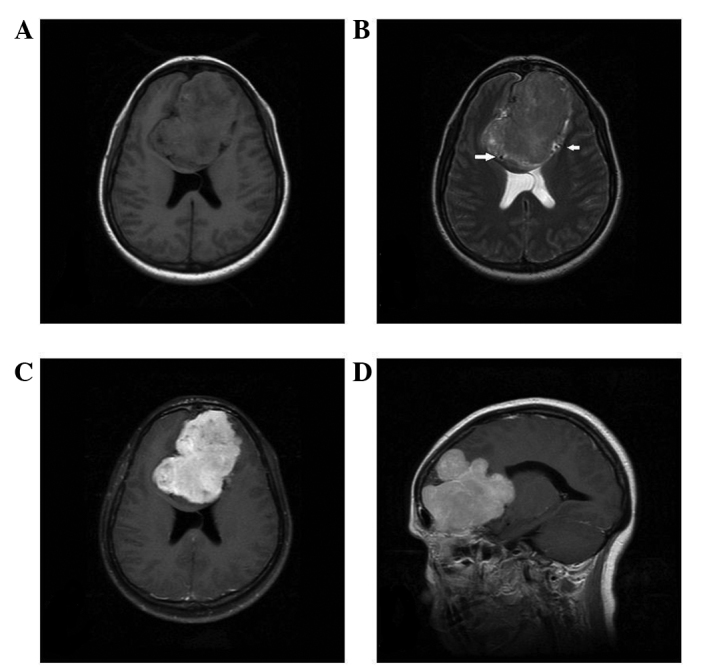
Patient 4. (A) Unenhanced magnetic resonance imaging scan reveals that the lesion is located in the anterior skull base with a well-defined border and lobulated shape. (B) The lesion exhibits a crow-midline growth pattern and intratumoral vessels are visible (arrow). (C) Enhanced scan reveals that the lesion is heterogeneously enhanced. (D) A narrow-based attachment to the skull base may be observed.

**Figure 5:**
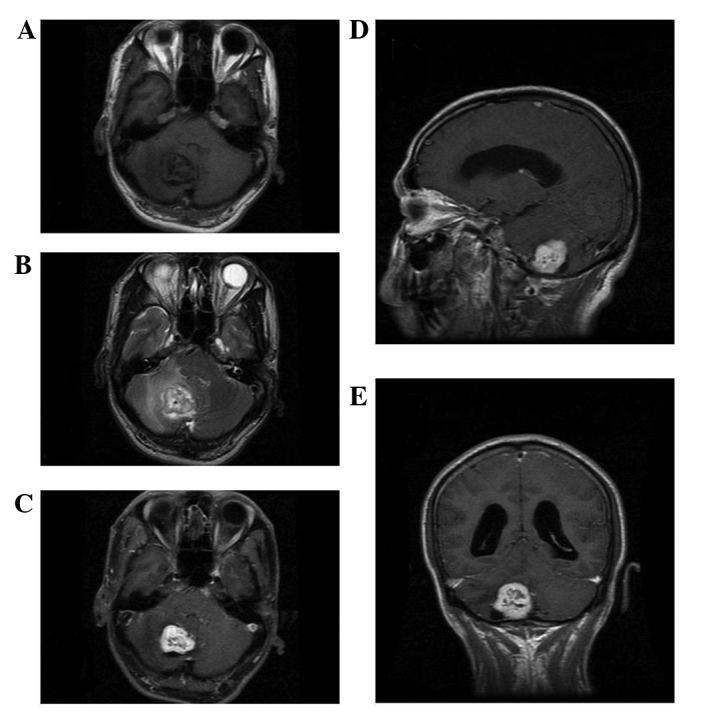
Patient 5. (A) Unenhanced magnetic resonance imaging scan reveals that the lesion is located in the infratentorial posterior fossa. (B) Peritumoral brain edema may be observed, and the fourth ventricle exhibits compressive change. (C) The lesion is markedly enhanced following injection of contrast agent. (D) The dural tail sign cannot be found, however, a (E) narrow-based attachment is visible and the intratumoral vessel may be detectable.

All cases were misdiagnosed as meningioma prior to surgery. MRI revealed that all five cases had a single lesion (in four cases located above the tentorium cerebelli; in one case located under the tentorium cerebelli). The lesions were lobular, measuring 3.0 to 7.5 cm with an iso-intense signal in T1WI and a slightly long signal in T2WI on the unenhanced MRI scan (Figs. 1–5). Four cases presented with a cross-midline growth pattern (Figs. 1, 3–5) and one case presented with a cross-lobe growth pattern (Fig. 2). One case exhibited dilatation of the lateral ventricle as the tumor compressed the fourth ventricle (Fig. 5). The adjacent bone was destroyed in one case (Fig. 2). Following injection of Gd-DTPA, no cases were found to exhibit the dural tail sign. Heterogeneous enhancement was observed in all cases (Figs. 1–5). Cystic degeneration, necrosis as well as flow void were observed in all cases (Figs. 1–5). The detailed MRI findings are listed in Table II.

### Pathological and immunohistochemical findings

**Figure 6:**
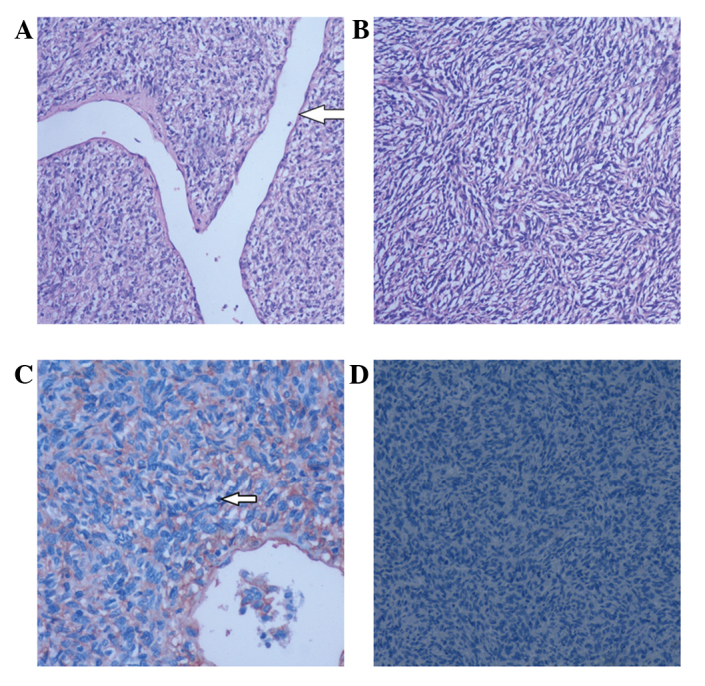
Patient 3.Pathological features of intracranial hemangiopericytoma. (A) On microscopic examination, tumor cells exhibit a diffuse growth pattern with abundant slit-shaped vessels in the central area (arrow). (B) The tumor cells are uniform in size with obscured nucleoli. (C) The nuclei of the tumor cells are oval and mitotic figures are occasionally observed (arrow). Immunohistochemical analysis reveals (C) marked positive expression of cluster of differentiation 34, but (D) negative expression of epithelial membrane antigen [stain, (A and B) hematoxylin and eosin, (C) cluster of differentiation 34, (D) epithelian membrane antigen; magnification, A, ×100; B, ×100; C, ×200; D, ×200].

Upon gross examination, the cut surfaces of the tumors were gray in color and fish-like in texture. The boundaries were clear with a complete or incomplete capsule. On microscopic examination, the tumor cells were shown to exhibit diffuse growth with abundant slit-shaped vessels in the central area. The cells were of uniform size with obscured nucleoli (Fig. 6A and B). The nuclei of the tumor cells were oval and mitotic figures were occasionally observed (Fig. 6C). No case exhibited the intranuclear inclusions that are relatively specific to meningioma. Calcification was only found in one case. Immunohistochemical analysis revealed a marked positive expression of CD34 (Fig. 6C), CD99 and Vim but negative expression of EMA (Fig. 6D), S100 and GFAP. Proliferating cell nuclear antigen Ki-67 immunohistochemical staining revealed that <5% of cells expressed Ki-67 in two cases and 5–10% of cells expressed Ki-67 in three cases. Syn staining revealed no expression in all cases.

## Discussion

Intracranial HPC is a rare tumor with aggressive behavior. HPC is usually known to occur in the musculoskeletal system and has been less frequently reported to occur in the central nervous system. The morbidity of HPC accounts for <1% of all intracranial tumors and ~2–4% of all meningeal tumors, worldwide (7). Owing to the unknown origin, HPC was hypothesized to originate from the meninges and thus was previously considered to be a subtype of meningioma. However, HPC is currently hypothesized to originate from Zimmermann pericytes (8).

Intracranial HPC usually occurs more commonly in males than females and the average age of presentation in patients with HPC was identified to range between 38 and 42 years in a previous series (9), which is similar to the findings of the present study. The symptoms of this type of tumor are non-specific and, furthermore, are similar to those of other types of tumors, such as meningeal meningoma (10). In general, HPC symptoms depend on the tumor size, extent and position. In the present cohort, the main symptoms were headache (n=5), dizziness (n=4), vomiting (n=2), weakness (n=1) and blurred vision (n=1). Routine laboratory tests revealed no specific findings.

On gross pathological examination, the lesion may manifest as a solitary nodule with a complete or incomplete capsule. In a study conducted by Zhou *et al* (11) examining 39 cases intracranial HPC and anaplastic HPC, the majority of the anaplastic HPC cases presented with an incomplete capsule and ill-defined boundary, but intracranial HPC had a complete capsule and clear boundary. The results of the present study are similar to those findings. Another previous study demonstrated that HPC more commonly occurs in the frontoparietal region (12). However, the present study observed no specific tumor location in all patients.

Intracranial HPC has a rich blood supply; marked heterogeneous enhancement was detected in the cases in the present study, which may also be explained by the pathological characteristics. On microscopic examination, the tumor cells exhibited diffuse growth patterns with abundant slit-shaped vessels. Intratumoral vessels were detected in all cases, which were indicated by flow voids on the MRI scans. This feature may be characteristic of HPC. In the present study, mitotic figures were occasionally detected. This feature indicates that intracranial HPC exhibits an aggressive behavior that results in recurrence and metastasis.

A previous study reported that HPC cells were strongly immunopositive for Vim, but negative for EMA, with CD34 expression focally positive and the endothelial cells always positive for CD34 (13). The results of the present study concurred with these findings.

To produce a correct preoperative diagnosis of intracranial HPC is difficult. In the present study, all cases were misdiagnosed as meningioma prior to surgery. Furthermore, the MRI features of HPC appear similar to those of meningioma. However, certain specific signs of intracranial HPC that are different from those of meningioma were identified in the present study. For example, flow void appears to be more common in intracranial HPC than in meningioma, as intracranial HPC has a richer blood supply and abundant slit-shaped vessels. In the present study, the growth patterns of intracranial HPC were as follows: Crossing the midline (n=4) and crossing the lobe (n=1) with a lobulated shape, which indicated that the intracranial lesions exhibited an invasive growth pattern. Compared with intracranial HPC, the growth pattern of meningioma appears to be more localized and the shape more regular. Furthermore, intracranial HPC exerts a destructive effect on the adjacent bone, unlike meningioma, which exerts a hyperplastic effect (14). This feature indicates that intracranial HPC exhibits a marked propensity for invasiveness. In addition, no case exhibited the dural tail sign in the present study. A previous study reported that dural tail sign was associated with the long-term response to the stimulation of the meninges by the tumor (15). Intracranial HPC is classified as WHO Grade II or III, and exhibits a rapid tumor growth rate and high malignant characteristics, therefore the dural tail sign is less common. Furthermore, a narrow dural attachment is another feature that differentiates intracranial HPC from meningioma (16). Intracranial HPC exhibits a narrow dural attachment, which is due to the malignant behavior of the tumor. However, meningioma commonly has a wide dural attachment.

Surgical resection of the tumor is the primary treatment choice in order to obtain a definitive diagnosis as well as to relieve symptoms (4). A cohort study conducted by Kumar *et al* (17) suggested that the main therapy for intracranial HPC was gross total resection combined with postoperative radiotherapy. In the present study, all cases underwent surgical resection combined with radiotherapy. In the follow-up period, only one case recurred within four years. Thus, a long-term follow-up is reasonable for the timely detection of recurrence.

In conclusion, intracranial HPC exhibits particular characteristics of WHO Grade II or III tumors, which are similar to those of meningioma. However, certain features may aid in differentiating intracranial HPC from meningioma. The flow void is a relatively specific sign common in intracranial HPC due to the rich blood supply. The growth pattern of intracranial HPC appears to be irregular with a lobulated shape. Adjacent bone erosion may also occasionally be identified in patients with intracranial HPC. In addition, a narrow dural attachment suggests a diagnosis of intracranial HPC rather than meningioma. Nevertheless, imaging alone should not be used to diagnose intracranial HPC; pathological examination is required for confirmation.

## Figures and Tables

**Table I:** Clinical findings from the five intracranial hemangiopericytoma cases.

Patient	Age	Sex	Recurrence	Headache	Dizziness	Vomiting	Weakness	Blurred vision
1	48	Male	No	Yes	Yes	Yes	No	Yes
2	60	Male	Yes	Yes	Yes	No	Yes	No
3	56	Female	No	Yes	No	No	No	No
4	37	Female	No	Yes	Yes	No	No	No
5	41	Male	No	Yes	Yes	Yes	No	No

**Table II:** Magnetic resonance imaging findings from the five intracranial hemangiopericytoma cases.

	Patient				
	
Tumor characteristic	1	2	3	4	5
Size	4.1 cm	5.3 cm	4.0 cm	7.5 cm	3.0 cm
Margin	Well-defined	Ill-defined	Well-defined	Well-defined	Well-defined
Morphology	Lobulated	Lobulated	Lobulated	Lobulated	Lobulated
Location	Right occipital region	Left temporal region	Left occipital region	Anterior skull base	Infratentorial posterior fossa
Growth pattern	Cross-midline	Cross-lobe	Cross-midline	Cross-midline	Cross-midline
Enhancement pattern	Heterogeneous	Heterogeneous	Heterogeneous	Heterogeneous	Heterogeneous
Bony destruction	No	Yes	No	No	No
Dural tail sign	No	No	No	No	No
Narrow-based attachment	Yes	Yes	Yes	Yes	Yes
Intratumoral vessels	Yes	Yes	Yes	Yes	Yes

## Abbreviations

HPC: hemangiopericytoma
MRI: magnetic resonance imaging
Vim: vimentin
EMA: epithelial membrane antigen
GFAP: glial fibrillary acidic protein
WHO: World Health Organization
